# Factors associated with dental clinic use by clients in Nigeria during the COVID-19 pandemic

**DOI:** 10.1186/s12903-024-04566-2

**Published:** 2024-07-17

**Authors:** Yewande Isabella Adeyemo, Mahsa Karimi, Elizabeth Oziegbe, Bamidele Olubukola Popoola, Michael Alade, Ahmad Reza Shamshiri, Heikki T. Murtomaa, Tosin Olajide Oni, Joycelyn Odegua Eigbobo, Nneka Kate Onyejaka, Olubukola Olamide Olatosi, Chioma Love Nzomiwu, Abdulrahman Bala Malami, Nneka Maureen Chukwumah, Olabimpe Abigail Soyoye, Mohammad Reza Khami, Morẹ́nikẹ́ Oluwátóyìn Foláyan

**Affiliations:** 1 Department of Child Dental Health, Bayero University, Kano, Kano State Nigeria; 2 Department of Community Health, Tehran University of Medical Sciences, Tehran, Iran; 3 Department of Child Dental Health, Obafemi Awolowo University, Ile-Ife, Nigeria; 4 Department of Child Oral Health, University of Ibadan, Ibadan, Nigeria; 5 Research Center for Caries Prevention, Dentistry Research Institute, Tehran University of Medical Sciences, Tehran, Iran; 6 Department of Oral Public Health, Institute of Dentistry, University of Helsinki, Helsinki, Finland; 7 Department of Demography and Social Statistics, Obafemi Awolowo University, Ile-Ife, Nigeria; 8 Department of Orthodontics and Paediatric Dentistry, Faculty of Dentistry, University of Port Harcourt, Port Harcourt, Nigeria; 9 Department of Child Dental Health, Faculty of Dentistry, University of Nsukka Teaching Hospital, Enugu, Nigeria; 10 Department of Child Dental Health, Faculty of Dental Sciences, College of Medicine, University of Lagos, Lagos, Nigeria; 11 Department of Child Dental Health, Faculty of Dentistry, College of Medical Sciences, University of Calabar, Calabar, Nigeria; 12 Department of Preventive Dentistry, School of Dentistry, College of Medical Sciences, University of Benin, Benin City, Nigeria; 13 Department of Child Dental Health, University of Medical Sciences, Ondo-City, Nigeria; 14 Oral Health Initiative, Nigerian Institute of Medical Research, Yaba, Lagos Nigeria; 15 Africa Oral Health Network, Alexandria University, Alexandria, Egypt

**Keywords:** Dental service utilization, COVID-19, Emergency dental treatment, Fear, Dental setting, Mass media

## Abstract

**Background:**

Nigeria, like many other countries, faced challenges in dental care provision during the COVID-19 pandemic, necessitating limited care to emergencies only. However, the impact of restricted access to dental services on oral health remains a concern, particularly w﻿ith preventive maintenance care. This study aims to identify the factors associated with dental service utilization during the COVID-19 pandemic among patients attending dental clinics in Nigeria, and their sources of information about COVID-19.

**Methods:**

This cross-sectional study recruited 500 participants who had visited dental clinics before and during the pandemic. Data were collected through telephone interviews and online questionnaires betw﻿een September and December 2021. Inferential analyses were conducted using ordered logistic regression models to assess the association between the independent (awareness of emergency dental treatments, knowledge of COVID-19 transmission routes in dentistry, awareness of required preparations in dental practice settings, fear of COVID-19 infection in dental settings), and dependent (utilization of dental services during the pandemic) variables after adjusting for confounding variables (age, sex, educational status, COVID-19 vaccinations status and COVID-19 status of live-in residents). The sources of COVID-19 information by dental patients was also identified. Statistical significance was established at 95% confidence level with a p value < 0.05.

**Results:**

Many participants were males (51.2%) and had tertiary education (61.6%). Fear of COVID-19 infection in dental settings was significantly associated with lower odds of utilizing dental care services during the pandemic (Adjusted Odds Ratio: -0.06, 95% Confidence Interval: -0.08, -0.01). The main sources of COVID-19 information were mass media and social networks, with only 42% receiving information from dental professionals.

**Conclusion:**

Fear of COVID-19 infection in dental settings significantly influenced dental service utilization during the pandemic. Strategies to address fear and improve communication channels between dental professionals and patients are essential for promoting oral health during similar crises.

## Introduction

Nigeria was one of the many countries affected by the COVID-19 pandemic. One of the immediate public health responses was limiting the provision of dental care services to emergency dental care and releasing a clinic procedural guidance document [1–3]. While this measure was important to give the dental clinics time to re-structure clinics physically and procedurally to reduce the risk of COVID-19 transmission between patients, and between patients and health care workers in the dental clinics [3–6], the limited access to dental care was a huge concern to the practicing dental healthcare workforces. Delaying access to preventive maintenance care was likely to result in a deterioration of oral health status.

There are multiple studies that suggest that limiting access of clients to the dental clinic during the pandemic proved to be detrimental to the oral health of clients. First, the pandemic affected several aspects of the oral health of individuals including limiting access to care when clients considered it necessary [7]. Second, COVID-19 infection was associated with dry mouth, oral lesions, orofacial pain, periodontal symptoms [8] and a decline in oral health quality of life [9, 10]. These oral health problems were poorly addressed during the pandemic and were not considered emergency care that required a dentist’s attention. Third, clients who needed prescribed emergency dental care were also kept away from the dental clinic, some because of poor awareness about the guidance protocol [11], and some may be because they were concerned about contracting COVID-19 from the dental clinic.

The studies on COVID-19 and oral health in Nigeria had been limited to dentists’ understanding of COVID-19 [12]. There was a study that identified that fear of contracting COVID-19 infection was the main reason for the unwillingness of clients to utilize dental services for routine care and oral prophylaxis in Nigeria [13]. There is little known about what factors may have made patients’ fear of contracting COVID-19 infection a critical/debilitating factor that limits their use of the dental clinic. Possible influencing factors include sociodemographic variables like age [14, 15], sex [15–17] and socioeconomic status [18, 19]; sources of information about COVID-19 [20]; awareness about dental clinic services being limited to emergency dental care during the pandemic [1]; awareness about the possible transmission of COVID-19 in dental setting [21]; or limited access to information about required preparations in the dental practice to prevent COVID-19 transmission [6].

The present study was designed using the Capabilities-Opportunities-Motivation-for-Behavior Assessment Framework (COM-B) [22] to understand the use of dental services during a pandemic. Studies have shown that dental service utilization in Nigeria is generally low [23–25]. Dental service utilization is an important health behavior that is synonymous with positive preventive dental care [26]. Understanding what factors were associated with the use of dental services during the COVID-19 pandemic by patients who are users of the dental clinic in Nigeria is a requisite for the design of effective and efficient behavioral interventions during future pandemics like that of COVID-19. The aim of the study was to assess the association between awareness of emergency dental treatments, knowledge of COVID-19 transmission routes in dentistry, awareness of required preparations in dental practice settings, fear of being infected with COVID-19 in dental settings and utilization of dental services during the COVID-19 pandemic. In addition, the study would determine the sources of information about COVID-19 among dental patients.

## Methods

### Ethical considerations

This study received approval from the Institute of Public Health Research Ethics Committee, Obafemi Awolowo University, Ile-Ife, Nigeria. This ethics committee served as the Ethics Committee of Records. Ethical clearance was also obtained from the Research Ethics Committee of Aminu Kano Teaching Hospital (NHREC/28/01/2020/AKTH/EC/3177), Health Research Ethics Committee, University of Calabar (UCTH/HREC/33/555), and University of Port Harcourt Teaching Hospital Research Ethics Committee (UPTH/ADM/90/S.II/VL.XI/1114). All study participants gave written consent for study participation.

### Study design, study participants and study settings

This was a cross-sectional study that recruited participants from a population of clients who had visited the dental clinic before and during the COVID-19 pandemic years. For this study, capacity was defined as an individual’s psychological and physical capacity to engage in the behavior of concern. Physical capability was concerned with whether an individual possesses the necessary knowledge and skills required to perform the target behavior. Psychological capability refers to an individual’s capacity to engage in the necessary thought processes, comprehension, and reasoning to perform the target behaviour [22]. For these reasons, the study recruited participants who had a history of dental hospital visits. Study participants had to have visited the dental clinic, be 18 years and older, and must have given consent to participate in the study either through face-to-face contact, telephone interview or responding to an online questionnaire link. There were no exclusion criteria. Study participants were recruited between 11th of September 2021 and 9th of December 2021 from tertiary dental hospitals located in Benin, Calabar, Enugu, Ibadan, Ile-Ife, Kano, Port-Harcourt, Lagos, and Ondo.

### Sample size

A convenient sample of 377 respondents was considered adequate for this study based on a margin of error of 5%, a 95% confidence interval and response distribution of 50%. The sample size for the study was rounded up to 380.

### Study participants recruitment

Participants were selected by systematic random sampling technique. First, the details of the patients who presented to the dental clinics between 2019 and 2021 were extracted from the medical records. A sample of participants from the study was selected through stratified random sampling. First, participants were stratified into years 2019, 2020 and 2021. Then, 190 patients were randomly selected from the 2019 list, 95 from the 2020 list and 95 from the 2021 list based on the relative number of patients who visited the clinic in each of these years. A stratified random selection of clients was done to include the similar number of patients from pre- and post-COVID-19 periods.

The lead investigators at each study site (NC, CN, NO, BP, MF, YA, JE, OO, OS) contacted each randomly selected study participant through a phone call to explain the purpose of the study and highlight data protection procedures. Participants who were willing to participate in the study set a date for the telephone interview or had access to a link to the online questionnaire through Telegram or WhatsApp. The online questionnaire was uploaded to Google Forms and the settings made the responses confidential, respondents untraceable, and could only submit once. Participants gave their consent by confirming the items on the first page of the form.

### Study independent variables

The study had four independent variables: (1). Awareness of emergency dental treatments, (2). Knowledge of COVID-19 transmission routes in dentistry, (3). Awareness of required preparations in dental practice settings, and (4). Fear of being infected with COVID-19 in dental settings.

Respondents were asked about awareness of emergency dental treatments by asking: “*Which of the following would you consider to be emergencies that require dental visit during the Corona virus pandemic?”* The nine responses, from which the respondents could choose multiple responses from were: (1) Painful swelling in or around your mouth and cheek; (2) Regular visits for braces and fixed orthodontic appliances (devices used in dentistry that align and straighten teeth and help position them with regard to a person’s bite); (3) Treatment of cavities that aren’t painful; (4) Regular visits for exams, cleanings, and x-rays; (5) Denture adjustment for people under radiotherapy and/or chemotherapy; (6) Broken or knocked out tooth; (7) Gum infection with pain or swelling; (8) Pain in a tooth, teeth or jawbone; and (9) Any lesion (painful or not) in the mouth persisting for two weeks or more. A tick for any of the responses was an indication that respondents considered the oral health problem an emergency that required dental visit during the COVID-19 pandemic.

Respondents were asked about their knowledge of COVID-19 transmission routes in dentistry by asking: *Which of the following can potentially transmit COVID-19 infection in dental settings* (you can choose more than one statement)? Respondents had eight options to choose from. These were; (1) Inhaling respiratory droplets produced when an infected person coughs, sneezes, or talks; (2) Through intact skin (healthy skin in which there are no breaks, scrapes, bruises etc.); (3) Contact with contaminated dental instruments and/or environmental surfaces; (4) Talking without a mask; (5) Close contact with other asymptomatic patients; (6) Inhaling fine droplets formed during dental procedures; (7) Contacts of oral, nasal, and eye mucous membrane with surfaces contaminated with oral fluids of other patients; and (8) Being in close contact (less than 2 m or 6 feet distance) with an infected person (patients, dentist, staff) for more than 15 min in a closed space without mask.

Respondents were asked about their awareness of required preparations in dental practice settings by asking: *Which of the following preparations against COVID-19 transmission are necessary in dental offices and clinics* (you can choose more than one statement)? Respondents had 11 options to choose from namely: (1) Adequate air conditioning (air flow or air circulation) in both waiting rooms and treatment room; (2) Screen staff and patients for flu-like symptoms, respiratory distress or fever as daily routine before going into the office/clinic; (3) Ensure a spatial sitting distance of at least 6 feet (2 m) among patients in the waiting area; (4) Enforce isolation of patients infected with/suspected of Corona virus infection in the waiting area; (5) Limit (or restrict) dental treatments for those infected with/suspected of Corona virus to only emergency procedures; (6) Use effective pre-procedural antimicrobial mouthwash; (7) Clean and disinfect public areas frequently, including door handles, chairs, and bathrooms; (8) Post visual alerts/ signs of instructions for hand and respiratory hygiene, and cough etiquette; (9) Use of full personal protective equipment including masks, gloves, gowns, and goggles or face shields by dentists and nurses; (10) Instruct patients to call before their appointment if they have respiratory symptoms so that staff can be prepared to care for them when they arrive; and (11) Complete tele/online evaluation before the first visit.

Respondents were also asked about fear of being infected with COVID-19 in dental settings. This was measured by nine questions with responses on a Likert-type scale with scores ranging from 0 (disagreed) to 10 (agreed). The questions assessed their beliefs, attitudes, and behavior about dental service access during the COVID-19 pandemic. The nine questions were: (1) During the Corona virus epidemic, I will not go to the dentist even when I have an emergent dental problem (e.g. pain, bleeding, and swelling); (2) During the Corona virus epidemic, the risk of infection with Corona virus is high in the dental field regardless of necessary preparations; (3) I am worried about getting Corona virus from my dentist; (4) I am worried Corona virus would be a long-term public health issue for human beings; (5) I am worried about transmitting Corona virus to my dentist in case I am infected and asymptomatic; (6) I avoid/delay dental visits because of the risk of infection from dental equipment; (7) I am worried about getting Corona virus infection from previous patients of the office; (8) I am worried about transmitting Corona virus to my loved ones in the case that no safety precautions were taken at the dental office; and (9) I am worried about getting Corona virus from the waiting room of the dental office.

### Study dependent variables

The dependent variable was the use of dental services during the COVID-19 pandemic by the study population. The use of the dental service was assessed by asking the question: *Have you visited a dental care clinic for consultation*,* either for treatment or routine checkup*,* at any time during the COVID-19 pandemic?* Respondents had one of five options to tick off. These were: The responses to the question were weighted as yes, I did for a routine visit, but I had no dental problem (5), yes, I did because of a non-emergency problem (4), yes, I did because of an emergency dental problem (3), no, I did not because I had no dental problem (2) and no, I did not visit, even though I had a dental problem (1). The outcome thus became a 5-level categorical variable with true order ranked from 5, the highest level of utilization (accessing routine care without any problem) to 1, the lowest level (refusing to access care despite having problem).

### Study confounding variables

The study confounding variables were divided into the background characteristics of participants and the COVID-19 related variables. The background characteristics were age at last birthday, sex (male or female), and educational status (no formal education, primary school only, secondary school only, Qur’anic school, diploma, bachelor’s, master’s, doctoral and postdoctoral degrees. Diplomas, bachelor’s, master’s, doctoral and postdoctoral degrees were categorized as tertiary education).

The COVID-19 related variables were the COVID-19 vaccination status, COVID-19 risk profile of live-in residents of the respondents and the COVID-19 testing status of the respondents. Respondents were also asked about their COVID-19 vaccination status. The question was: Have you been vaccinated against the Corona virus? Respondents could tick either a “yes” or “no” response. A ‘yes’ response indicated that the study participant was vaccinated. A ‘no’ response indicated that the study participant was not vaccinated.

Respondents were also asked about the COVID-19 status of live-in residents. Respondents could pick one of three options namely: living with at-risk people, living with people with no known risk; and living alone. In addition, respondents were asked if they had ever tested positive for COVID-19. They had two response options: Never tested positive, tested positive at least once.

### Questionnaire validation

The questionnaire was developed and validated through an extensive process [27]. Articles on knowledge about COVID-19 or other airborne diseases such as SARS and dental fear in similar situations were identified through electronic search of PubMed/MEDLINE, Google Scholar, Scopus, and Web of Science. The identified reference materials were used to develop the closed-ended study questionnaire, following Stehr-Green scale [28]. Domain generation was performed via a deductive and inductive approach. A group of experts in the fields of dental public health and epidemiology developed a set of possible domains of interest [29].

The questionnaire was first developed in English. A content validation of the questionnaire was done by eight experts in community dentistry (six bilingual experts and two native English speakers). The experts rated each of the items of the questionnaire for essentiality, relevance, clarity, and simplicity. Each item was rated on a scale of 1–3 for essentiality and 1–4 for relevance, clarity, and simplicity respectively. Experts were also asked to make comments and suggestions for improving the face validity of the questionnaire and this was used to modify the wording of some items of the questionnaire. The expert ratings were used to analyze for the content validity of the questionnaire by calculating Item Content Validity Index (I-CVI) and Scale Content Validity Index (S-CVI/Ave) [30]. An I-CVI score of less than 0.86 was considered as a threshold for removing items from the questionnaire [31]. The I-CVI scores ranged from 0.29 to 1 and the S-CVI/Ave, ranged from 0.62 to 0.99. Four items were removed, due to I-CVI score for essentiality of less than 0.86. Two items with similar interpretations were merged. In addition, 14 items were modified for language clarity.

The revised English version of the questionnaire was sent to 60 English speaking respondents, resident in Nigeria and the United States who filled the questionnaire and made comments and suggestions on how to make phrases simple and comprehensible. Cronbach’s alpha analysis was conducted to assess the reliability of sections of the questionnaire. The average completion time was also recorded. The Cronbach’s alpha for the sections on awareness of emergency dental treatments, knowledge of COVID-19 transmission routes in dentistry, awareness of required preparations in dental practice settings and fear of being infected with COVID-19 in dental settings were 67.5%, 70.7%, 83.3% and 85.1% respectively.

### Data analysis

All the responses from Google Forms were downloaded to a Microsoft Excel 2013 file. The cleaned and coded data was analyzed using Stata 16. A descriptive analysis was conducted for the dependent, independent, and confounding variables. Frequency and percentage distribution were used to describe the categorical variables and mean analysis was used for the numeric variable. For the dependent variables, the number of correct responses given was weighted and cumulated to generate a score for each respondent.

The measure of central tendency was used to divide the scores into low, medium, or high levels. Previous studies have used similar methodologies to disaggregate numeric scores into categories based on the central tendencies of the scores [32, 33].

For ‘awareness of emergency dental treatments’, scores ranged from 11 to 100 with a mean of 65 ± 18. Scores from 11 to 47 were grouped as ‘low awareness’; scores from 47.01 to 83 were grouped as ‘moderate awareness’ while scores from 83.01 to 100 were grouped as ‘high awareness’.

For ‘knowledge of COVID-19 transmission routes in dentistry’, the scores ranged from 12 to 100 with a mean of 61 ± 22. Scores from 12 to 39 were grouped as ‘low knowledge’, scores from 39.01 to 83 were grouped as ‘moderate knowledge, and scores from 83.01 to 100 were grouped as ‘high knowledge’.

For ‘awareness of required preparations in dental practice settings’, the scores ranged from 0 to 100 with a mean of 65 ± 18. Scores from 0 to 47 were grouped as low awareness of required preparations in dental practice settings, scores from 47.01 to 83 were grouped as ‘moderate awareness’ and scores from 83.01 to 100 were grouped as ‘high awareness’.

The scores for ‘fear of being infected with corona virus in dental settings’ ranged from 0 to 100 with a mean of 55 ± 17. Scores from 0 to 38 were grouped as ‘low fear’, scores from 38.01 to 72 were grouped as ‘moderate fear’ and scores from 72.01 to 100 were grouped as ‘high fear’.

Respondents were also asked about their source of information on COVID-19 by asking: *From which source do you receive information regarding COVID-19* (you can choose more than one statement)? Respondents had seven options to choose from. The findings were described using a bar chart.

A test of multicollinearity was performed on the variables to examine if any collinear variable that could affect the reliability of the regression slopes existed in the dataset. Variables with Variance Inflation Factor (VIF) above 5 were considered multicollinear [33]. However, all the study variables had VIF below 5, hence, none of them was dropped.

Ordered logistic regression (OLR) models were fitted to estimate the effects of awareness of emergency dental treatments, knowledge of COVID-19 transmission routes in dentistry, awareness of required preparations in dental practice settings, and fear of being infected with COVID-19 in dental settings on the utilization of dental care services during the COVID-19 pandemic because the variable had categorical levels with true order ranking ranging from 1 (failure to utilize service despite having a dental problem), to 5 (routine utilization without having any problem) [34]. The model was adjusted for the effects of the confounding variables and the adjusted odds ratio computed. Statistical significance was established at 95% confidence level with a *p* value < 0.05.

## Results

There were 500 participants recruited for the study—472 (94.4%) through telephone calls and 28 (5.6%) through the online platform. This number exceeded the proposed sample size by 31.1%. Table 1 shows the profile of the 500 respondents recruited into the study. The age of the study participants ranged from 18 to 89 years with a mean (standard deviation) age of 36.85 *±* 13.73. The study participants included 256 (51.2%) males and 308 (61.6%) who had tertiary education.

In addition, 79 (15.8%) had high level of awareness about emergency dental treatments, 105 (21.0%) had high knowledge of COVID-19 transmission routes in dentistry, 73 (14.6%) had high level of awareness about required preparations to prevent COVID-19 transmission in dental practice settings, and 83 (16.6%) had high level of fear of being infected with corona virus in dental settings. Furthermore, there were 164 (32.8%) who visited the dental clinic because of non-emergency dental problems, 333 (66.6%) who had not taken a COVID-19 vaccine and 291 (58.1%) who were living with people who had no known risk for COVID-19.

**Table 1:** Characteristics of study participants (*N* = 500)

**Age (years), M ± SD (range)**	36.85 ± 13.73 (18–89)
**Gender,** ***N*** **(%)**	
Female	244 (48.8)
Male	256 (51.2)
**Education level**,** N (%)**	
Up to Secondary	125 (25.0)
Associated Degrees	67 (13.4)
Bachelor	232 (46.4)
Higher than Bachelor’s	76 (15.2)
**Dental visits**,** N (%)**	
Yes, because of a non-emergent problem	164 (32.8)
Yes, because of an urgent problem	193 (38.6)
No, because of COVID-19, although I had some problems in my mouth	13 (2.6)
No, because I did not have any problem	130 (26.0)
**COVID-19 vaccinated**,** N (%)**	
Yes	167 (33.4)
No	333 (66.6)
**COVID-19 risk in resident**,** N (%)**	
Living with at-risk people	91 (18.2)
Living with people with no known risk	291 (58.2)
Living alone	118 (23.6)
**COVID-19 status**	
Never tested positive	485 (97.0%)
At least one positive test	15 (3.0%)
**Level of awareness about emergency dental treatments**	
Low	104 (20.8)
Moderate	317 (63.4)
High	79 (15.8)
**Knowledge of COVID-19 transmission routes in dentistry**	
Low	140 (28.0)
Moderate	255 (51.0)
High	105 (21.0)
**Level of awareness about required preparations in dental practice settings**	
Low	146 (29.2)
Moderate	281 (56.2)
High	73 (14.6)
**Fear of being infected with corona virus in dental settings**	
Low	76 (15.2)
Moderate	341 (68.2)
High	83 (16.6)

Table 2 shows the inferential analysis on the associations between the dependent and independent variables. Awareness about emergency dental treatments, knowledge of COVID-19 transmission routes in dentistry, and level of awareness about required preparations in dental practice settings were not significantly associated with the utilization of dental care services during the COVID-19 pandemic. However, the fear of being infected with corona virus in dental settings was significantly associated with the utilization of dental care services during the COVID-19: participants who had the fear of being infected with the virus had significantly lower odds of utilizing the dental care services during the pandemic (AOR: -0.06; 95% CI: -0.08, -0.01).

**Table 2:** Factors associated with the utilization of dental services during COVID-19

Variables		Model
		AOR (95% C.I)
**Background characteristics**		
Gender	Female	Ref
	Male	0.25 (0.13, 0.33)*
Education	Up to secondary	Ref
	Associated degrees	0.38 (0.32, 1.21)
	Bachelor	1.04 (0.81, 1.12)
	Higher	0.55 (0.42, 1.09)
Age		0.003 (0.001, 0.012)*
**COVID-related factors**		
COVID risk in residence	Living with at-risk people	Ref
	Living with people with no known risk	-0.28 (-0.53, 0.11)
	Living alone	0.67 (0.43, 1.12)
Vaccination status	At least one shot	Ref
	Not vaccinated	1.04 (1.03, 1.31)
COVID status	Never tested positive	Ref
	At least one positive test	-4.71 (-3.23, 0.24)
**Knowledge and awareness**		
Awareness about emergency dental treatments		-0.02 (-0.01, 0.04)
Knowledge of COVID-19 transmission routes in dentistry		0.02 (0.01, 1.09)
Level of awareness about required preparations in dental practice settings		0.02 (0.01, 1.23)
Fear of being infected with corona virus in dental settings		-0.06 (-0.08, -0.01)*

*Statistically significant association at *p* < 0.05

**Fig. 1:**
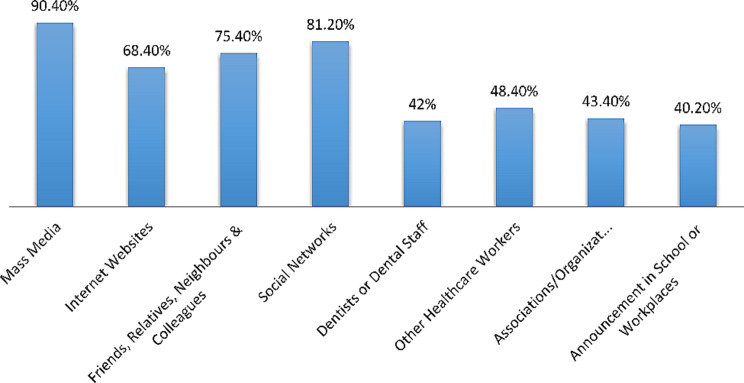
Sources through which respondents received information about coronavirus

Results on the sources through which the respondents received information about coronavirus are shown in Fig. 1. The main source of information was from the mass media and from social networks. Only 42% of participants received information about the coronavirus from the dentists and dental staff.

## Discussion

The findings of this study shed light on the factors influencing the utilization of dental services during the COVID-19 pandemic among patients attending dental clinics in Nigeria. The results indicated that as at the third wave of the COVID-19 pandemic in Nigeria, about 1 in every 5 patients or less who had attended the dental clinic in Nigeria were aware of emergency dental treatments and required preparations in dental practice settings, had knowledge of COVID-19 transmission routes in dentistry and had the fear of being infected with COVID-19 in dental settings. There were no statistically significant associations between the awareness of emergency dental treatments, knowledge of COVID-19 transmission routes in dentistry, the awareness of required preparations in dental practice settings and utilization of dental services during the COVID-19 pandemic. However, patients who had the fear of being infected with COVID-19 in dental settings seem less likely to have utilized the dental clinic during the COVID-19 pandemic.

One of the strengths of this study is its thorough examination of how the COVID-19-related characteristics of patients attending dental clinics in Nigeria influenced their utilization of dental services during the pandemic. This insight is valuable as it can inform future pandemic planning efforts for this specific subset of healthcare users. Additionally, the study benefited from being guided by a theoretical framework, enhancing the interpretative capability and contextual understanding of the study findings.

However, the study also had some limitations. First, its cross-sectional design precludes the establishment of cause-effect relationships. Secondly, the recruitment of study participants was limited to those accessible via phone or online platforms, potentially excluding certain dental service users, particularly those with lower socioeconomic status who may lack access to such technology. In addition, though the demographic profile of the study participants revealed a relatively diverse sample that potentially reflects a wide range of perspectives and experiences, the mean age of participants indicates that younger adults were more prominently represented in the study. Despite these limitations, the study findings provided some valuable insights into utilization of dental services during the COVID-19 pandemic.

First, the COM-B model views behaviour as a component within a network of interacting factors. It postulates that to observe a particular behaviour at a specific juncture, an individual must possess both the capacity and opportunity to enact the behaviour, with the motivation to engage in the behaviour outweighing that for any alternative behaviours [35]. Our study findings suggest that the non-use of the dental clinic during the COVID-19 pandemic by a population that had the capacity and the opportunity to visit the dental clinic based on their history of dental service utilization, was mainly driven by the fear of contracting COVID-19 in the clinic.

Patients who utilize the dental clinic in Nigeria are majorly those in need of curative care [25, 36, 37]. They therefore may have need for multiple dental visits to the clinic to complete their curative care needs and to institute preventive care. Disruption of access of these clients to the dental clinic by the pandemic creates the risk for deteriorating oral health [38, 39]. However, where fear worsens the prospect for dental service utilization, this intersection may worsen the risk for deteriorating dental health. Our study findings emphasize the need for targeted interventions aimed at alleviating patients’ fears and addressing misconceptions about the safety of dental care facilities during the pandemic. This may reduce the plausible risk for deteriorating oral health by those who need the services. Information to allay these fears could be relayed using mass media and through social networks campaigns as these are the two main sources of information about COVID-19 in this study population. The mass media is associated with increased access to unbiased information sources during pandemics [40, 41].

However, intolerance of uncertainty, depression, anxiety, and stress is associated with the fear of COVID-19 infection, and the fear of COVID-19 limits the ability of people to be positive [42, 43]. Thus, to address the fear of COVID-19, it is important to recognize the mediators and its impact on the strategies to prevent emergence of fear during pandemics like COVID-19 and the messaging on its impact on oral health positivity. The study results suggest that a large proportion of participants in this study had moderate to high levels of awareness about emergency dental treatments, knowledge of COVID-19 transmission routes in dentistry, and awareness of required preparations in dental practice settings. These findings underscore the importance of considering patients’ perceptions and concerns when designing strategies to improve dental service utilization during public health crises such as the COVID-19 pandemic.

This perception is reinformed by the theoretical framework for this study. Contrary to expectations, awareness about emergency dental treatments, knowledge of COVID-19 transmission routes in dentistry, and awareness of required preparations in dental practice settings were not significantly associated with dental service utilization. We perceive that having the capacity and the opportunity to visit the dental clinic (the expected enacted behaviour in this study) may not be enough to motivate people who use the dental clinic prior to the pandemic to use the clinic during the pandemic. The motivation to engage in the expected behaviour seems to outweigh that for any alternative behaviours [44]. There may be a need for gender considerations in designing strategies for dental service utilizations as a previous study had indicated that more males visited the dental clinic during the pandemic as they did not express their fears but rather, they emphasized strength and bravery hence visited the dental clinic more [42]. In addition, visits to the dental clinic during the pandemic was more symptomatic [36, 45].

In conclusion, this study underscores the importance of understanding patients’ perceptions, fears, and information-seeking behaviours in shaping dental service utilization during the COVID-19 pandemic. Addressing patients’ fears, improving communication between dental professionals and patients, and ensuring the provision of accurate information are essential steps towards promoting oral healthcare access and maintaining continuity of dental services during public health crises. Further research and targeted interventions are warranted to effectively address the challenges identified in this study and optimize dental service utilization in similar contexts.

## Data Availability

The data that support the findings will be available from the corresponding author upon request following a 6-month embargo from the date of publication. Requests will be controlled, and each request will be considered on a case-by-case basis.

## Abbreviations

AOR: Adjusted Odds Ratio
CI: Confidence Interval
COVID-19: Coronavirus Infectious Disease 2019
COM-B: Capabilities-Opportunities-Motivation-for-Behavior Assessment Framework

## References

[1] World Health Organization. World Health Organization advises delaying of non-essential dental care in areas of COVID-19 community transmission. 11 August 2020.

[2] Alharbi A, Alharbi S, Alqaidi S (2020). Guidelines for dental care provision during the COVID-19 pandemic. Saudi Dent J.

[3] Jiang CM, Duangthip D, Auychai P, Chiba M, Folayan MO, Hamama HHH (2021). Changes in oral Health policies and guidelines during the COVID-19 pandemic. Front Oral Health.

[4] Checchi V, Bellini P, Bencivenni D, Consolo U. COVID-19 dentistry-related aspects: a literature overview. Int Dent J. 2020; 1–7. 10.1111/idj.12601 PMC7361251 33616049

[5] Meng L, Hua F, Bian Z (2020). Coronavirus disease 2019 (COVID-19): emerging and future challenges for dental and oral medicine. J Dent Res.

[6] Kampf G, Todt D, Pfaender S, Steinmann E (2020). Persistence of coronaviruses on inanimate surfaces and their inactivation with biocidal agents. J Hosp Infect.

[7] Dickson-Swift V, Kangutkar T, Knevel R, Down S (2022). The impact of COVID-19 on individual oral health: a scoping review. BMC Oral Health.

[8] Qi X, Northridge ME, Hu M, Wu B (2022). Oral health conditions and COVID-19: a systematic review and meta-analysis of the current evidence. Aging Health Res.

[9] Knorst JK, Brondani B, Tomazoni F, Vargas AW, Cósta MD, da Silva Godois L, Mendes FM, Ardenghi DM, Ardenghi TM (2021). COVID-19 pandemic reduces the negative perception of oral health-related quality of life in adolescents. Qual Life Res.

[10] Avasthi A, Kalra T, Singh B (2022). Oral Hygiene practices and oral Health Related Quality of Life observed in patients reporting to Dental Institution in North India during COVID-19 pandemic. J Prev Med Hyg.

[11] Parvaie P, Osmani F (2022). Dentistry during COVID-19: patients’ knowledge and satisfaction toward health protocols COVID-19 during dental treatment. Eur J Med Res.

[12] Aladelusi TO, Atiba FA, Gbadebo SO, Adeyemo YI, Olusanya AA, Akadiri OA (2021). COVID-19 outbreak and dental health care provision in Nigeria: a national survey. BMC Oral Health.

[13] Akinloye SJ, Sopeju IF, Akinloye CU, Adegoke KT, Lawal FB (2021). Impact of COVID-19 pandemic lockdown on oral Hygiene practices and willingness to utilize Dental Services among Patient attendees of a Primary Oral Health Care Clinic in Ibadan, Nigeria. Nig Dent J.

[14] Yanex ND, Weiss NS, Romand JA, Tregiarri MM (2020). COVID-19 mortality risk for older men and women. BMC Public Health.

[15] Caycho-Rodríguez T, Tomás JM, Vilca LW, Carbajal-León C, Cervigni M, Gallegos M (2021). Socio-demographic variables, fear of COVID-19, anxiety, and Depression: Prevalence, relationships and Explanatory Model in the General Population of Seven Latin American Countries. Front Psychol.

[16] Kharroubi SA, Diab-El-Harake M (2022). Sex-difference in COVID-19 diagnosis, risk factors and disease comorbidities: a large US-based cohort study. Front Public Health.

[17] Cerda AA, García LY (2022). Factors explaining the fear of being infected with COVID-19. Health Expect.

[18] Fernandez-Martinez NF, Ruiz-Montero R, Gomez-Barroso D, Rodriguez-Torronteras A, Lorusso N, Salcedo-Leal I (2022). Socioeconomic differences in COVID-19 infection, hospitalization and mortality in urban areas in a region in the South of Europe. BMC Public Health.

[19] Ibrahim MS, Alibrahim H, Al Madani A, Alamri A, Bamashmous M, Tounsi A (2021). Fear factor in seeking Dental Care among saudis during COVID-19 pandemic. 2021. Int J Environ Res Public Health.

[20] Amara PS, Platt JE, Raj M, Nong P (2022). Learning about COVID-19: sources of information, public trust and contact tracing during the pandemic. BMC Public Health.

[21] González-Olmo MJ, Ortega-Martínez AR, Bendición DB, Romero-Maroto M, Carrillo-Diaz M (2020). Perceived vulnerability to coronavirus infection: impact on dental practice. Braz Oral Res.

[22] Michie S, van Stralen MM, West R (2011). The behaviour change wheel: a new method for characterising and designing behaviour change interventions. Implement Sci.

[23] Osuh ME, Oke GA, Asuzu MC (2014). Dental services and attitudes towards its regular utilization among civil servants in Ibadan, Nigeria. Ann Ib Postgrad Med.

[24] Sofola OO (2010). Implication of low oral health awareness in Nigeria. Niger Med J.

[25] Osadolor OO, Akaji EA, Otakhoigbogie U, Amuta HC, Obi DI, Osadolor AJ (2019). Dental Service utilization of a Rural Population in Nigeria. Int J Dent Res.

[26] Atchison KA, Mayer-Oakes SA, Schweitzer SO, Lubben JE, De Jong FJ, Matthias RE. The relationship between dental utilization and preventive participation among a well-elderly sample. J Public Health Dent. 1993 Spring;53(2):88–95. 10.1111/j.1752-7325.1993.tb02681.x. 10.1111/j.1752-7325.1993.tb02681.x 8515416

[27] Karimi M. Knowledge and attitude of dental patients regarding COVID-19 infection in dental settings. M.Sc. Thesis, Tehran University of Medical Sciences. 2022. https://lib.tums.ac.ir/site/catalogue/245627.

[28] Stehr-Green PA, Stehr-Green JK, Nelson A, Alexander L, Mejia GC, MacDonald PD (2003). Developing a questionnaire. FOCUS Field Epidemiol.

[29] Boateng GO, Neilands TB, Frongillo EA, Melgar-Quiñonez HR, Young SL. Best practices for developing and Validating Scales for Health, Social, and behavioral research: a primer. Front Public Health. 2018;6. 10.3389/fpubh.2018.00149. 10.3389/fpubh.2018.00149 PMC6004510 29942800

[30] Yusoff MSB (2019). ABC of Content Validation and Content Validity Index calculation. Educ Med J.

[31] Lynn MR (1986). Determination and quantification of content validity. Nurs Res.

[32] Manikandan S. Measures of central tendency: Median and mode. J Pharmacol Pharmacother. 2011;2(3):214–215. 10.4103/0976-500X.83300. PMID: 21897729. 10.4103/0976-500X.83300 PMC3157145 21897729

[33] Oni TO, Adebowale SA, Afolabi AA, Akinyemi AA, Banjo OO. Perceived health facility-related barriers and post-abortion care-seeking intention among women of reproductive age in Osun state, Nigeria. BMC Womens Health. 2023;23(311). 10.1186/s12905-023-02464-3. 10.1186/s12905-023-02464-3 PMC10273618 37328732

[34] Singh V, Dwivedi SN, Deo SVS. Ordinal logistic regression model describing factors associated with extent of nodal involvement in oral cancer patients and its prospective validation. BMC Med Res Methodol. 2020;20(95). 10.1186/s12874-02000985-1. 10.1186/s12874-020-00985-1 PMC7183690 32336269

[35] Michie S, West R, Campbell R, Brown J, Gainforth H (2014). ABC of behaviour change theories: an essential resource for researchers, policy makers and practitioners.

[36] Olusile AO, Adeniyi AA, Orebanjo O (2014). Self-rated oral health status, oral health service utilization, and oral hygiene practices among adult nigerians. BMC Oral Health.

[37] Folaranmi N, Akaji E, Onyejaka N (2014). Pattern of presentation of oral health conditions by children at university of Nigeria Teaching Hospital, Enugu: a retrospective study. Niger J Clin Pract.

[38] Ren YF, Rasubala L, Malmstrom H, Eliav E (2020). Dental Care and oral health under the clouds of COVID-19. JDR Clin Trans Res.

[39] Oluwatola TI, Olowookere OM, Folayan MO (2022). COVID-19 pandemic and the widening oral health inequality in Nigeria. Pan Air Med J.

[40] Mass Media Coverage Helps Slow Down Disease Spread in an Epidemic. Reports Should Focus on Changing Behaviour. Say Researchers. http://www.sciencedaily.com/releases/2016/01/160126130819.html.

[41] Yakunin K, Mukhamediev RI, Zaitseva E, Levashenko V, Yelis M, Symagulov A (2021). Mass Media as a Mirror of the COVID-19 pandemic. Computation.

[42] Bakio ˘glu F, Korkmaz O, Ercan H. Fear of COVID-19 and positivity: mediating role of intolerance of uncertainty, depression, anxiety, and stress. Int J Ment Health Addic 10.1007/s11469-020-00331-y. 10.1007/s11469-020-00331-y PMC7255700 32837421

[43] Hyland P, Shevlin M, McBride O, Murphy J, Karatzias T, Bentall RP (2020). Anxiety, and depression in the Republic of Ireland during the COVID-19 pandemic. Acta Psychiatr Scand.

[44] Reyna VF, Farley F, Risk (2006). Rationality in adolescent decision making: implications for theory, practice, and public policy. Psychol Sci Public Interest.

[45] Kumar U, Gupta A, Goyal A, Gauba K (2021). Impact of covid-19 pandemic on characteristics of dental emergencies and treatment services at tertiary care centre. Saudi Dent J.

